# Co-Occurrence of Rarest Type of Dysphagia Lusoria (Type N-1) and Eosinophilic Esophagitis in a Cognitively Disabled Individual

**DOI:** 10.1155/2019/2890635

**Published:** 2019-11-11

**Authors:** Kishore Kumar, Jasbir Makker, Hassan Tariq, Ariyo Ihimoyan, Chime Chukwunonso, Masooma Niazi, Michael Lombino, Muhammad Kamal, Harish K. Patel

**Affiliations:** ^1^Division of Gastroenterology, BronxCare Hospital Center a Clinical Affiliate of Mt Sinai Health Systems and Academic Affiliate of Icahn School of Medicine, Bronx, NY 10457, USA; ^2^Department of Medicine, BronxCare Hospital Center a Clinical Affiliate of Mt Sinai Health Systems and Academic Affiliate of Icahn School of Medicine, Bronx, NY 10457, USA; ^3^Department of Pathology, BronxCare Hospital Center a Clinical Affiliate of Mt Sinai Health Systems and Academic Affiliate of Icahn School of Medicine, Bronx, NY 10457, USA; ^4^Department of Radiology, BronxCare Hospital Center a Clinical Affiliate of Mt Sinai Health Systems and Academic Affiliate of Icahn School of Medicine, Bronx, NY 10457, USA

## Abstract

Dysphagia is an expressive symptom, described by an individual as “difficulty in swallowing.” Dysphagia due to esophageal compression from an aberrant right subclavian artery is rare, and it is termed as “dysphagia lusoria.” We present a rare case of co-occurrence of dysphagia lusoria with esophageal eosinophilia in a patient with cognitive disability which portends a case with diagnostic challenge and treatment dilemma. A 31-year-old man with intellectual disability, cerebral palsy, previous history of feeding difficulty, and esophageal food impaction presented with esophageal foreign body impaction. He has no known history of atopy and food allergies. There was no laboratory evidence of peripheral eosinophilia. The IgE-mediated allergic test was unremarkable. His prior presentation revealed a diagnosis of eosinophilic esophagitis. The imaging studies showed proximal esophageal dilatation with extrinsic compression at the level of the upper esophagus. The foreign bodies were removed successfully through the help of upper endoscopy. Subsequent evaluation revealed a rare type of dysphagia lusoria (type N-1) due to an aberrant left subclavian artery arising from the right-sided aortic arch. The patient's family refused further management of artery lusoria. Prolonged stasis of secretions and food in the esophagus can also lead to increased esophageal eosinophils. In our case, it remains undetermined whether increased number of esophageal eosinophils resulted from primary eosinophilic esophagitis or due to prolonged food stasis from esophageal compression caused by an aberrant subclavian artery. However, food impaction right above the compression site makes dysphagia lusoria the likely etiology.

## 1. Introduction

“Dysphagia,” defined as difficulty in swallowing, is an expressive and patient-reported symptom. Given the lack of expression, evaluation and diagnosis remain a challenge in a patient with cognitive disability. Throughout a lifetime, dysphagia is present in 80 to 90 percent of individuals with cognitive disability [1]. Dysphagia can present as a “feeding difficulty” given the lack of expression of symptoms, and this necessitates the Dysphagia Disorder Survey (DDS) assessment [1].

Dysphagia can originate from oropharyngeal, esophageal, and gastric pathology [2]. The esophageal etiology can be differentiated into mechanical, neuromuscular, or inflammatory conditions [3]. The mechanical conditions leading to dysphagia can be from intrinsic obstruction (mass or stricture) or extrinsic esophageal compression of mediastinal structures. Esophageal inflammatory conditions resulting in dysphagia include eosinophilic esophagitis that affects esophageal mucosa. The coexistence of multiple etiologies leading to dysphagia is extremely rare and not reported before.

We present a case of dysphagia in a cognitively disabled individual that presented as feeding difficulty and cough associated with food swallow. Workup revealed a diagnosis of eosinophilic esophagitis (EoE). Later, he was also found to have an aberrant left subclavian artery causing esophageal compression, as the cause of dysphagia. The coexistence of these two etiologies has never been reported. The extrinsic compression has not been shown as an etiology for secondary eosinophilic esophagitis [4]. Our case is unique as it demonstrates the rare congenital abnormality of the aberrant subclavian artery origin. Given the lack of history due to cognitive disability in our patient, this case highlights the hurdles posed in diagnosis and management of this patient.

## 2. Case Presentation

**Figure 1:**
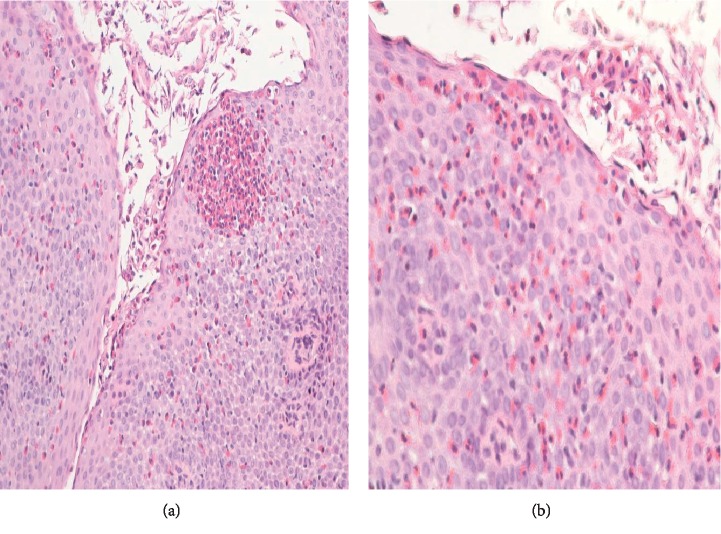
(a) Eosinophilic esophagitis with eosinophilic microabscess showing aggregates of mainly intact eosinophils in an area of mucosa associated with epithelial cell degeneration (H&E, magnification ×200). (b) Eosinophilic esophagitis showing numerous intraepithelial eosinophils (measuring >40 per high-power field) and accumulation of eosinophils in the superficial portion of the epithelium (H&E, magnification ×200).

**Figure 2:**
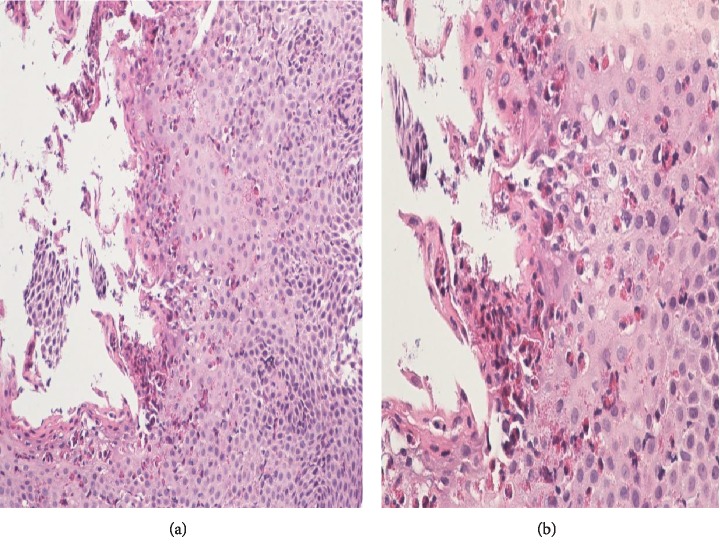
(a) Eosinophilic esophagitis showing accumulation of eosinophils within the superficial necroinflammatory debris. The intraepithelial eosinophils measure >30 per high-power field. There is marked reactive squamous hyperplasia (H&E, magnification ×200). (b) Eosinophilic esophagitis with eosinophilic microabscess and increased intraepithelial eosinophils measuring >30 per high-power field (H&E, magnification ×400).

**Figure 3:**
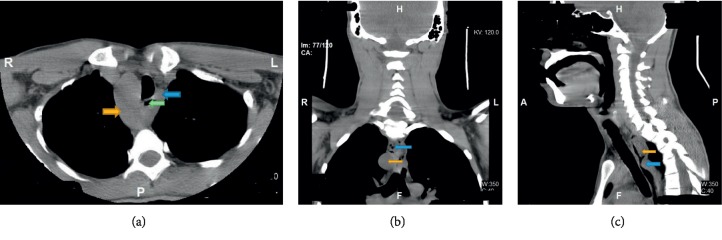
(a) Cross section of the chest with the right-sided aortic arch (yellow) with the subclavian artery (blue) enclosing the esophagus (green). (b) Coronal section of the chest and neck with esophageal compression due to the subclavian artery (yellow) and debris retention (blue). (c) Sagittal section of the chest and neck with esophageal compression due to the subclavian artery (yellow) and debris retention (blue).

**Figure 4:**
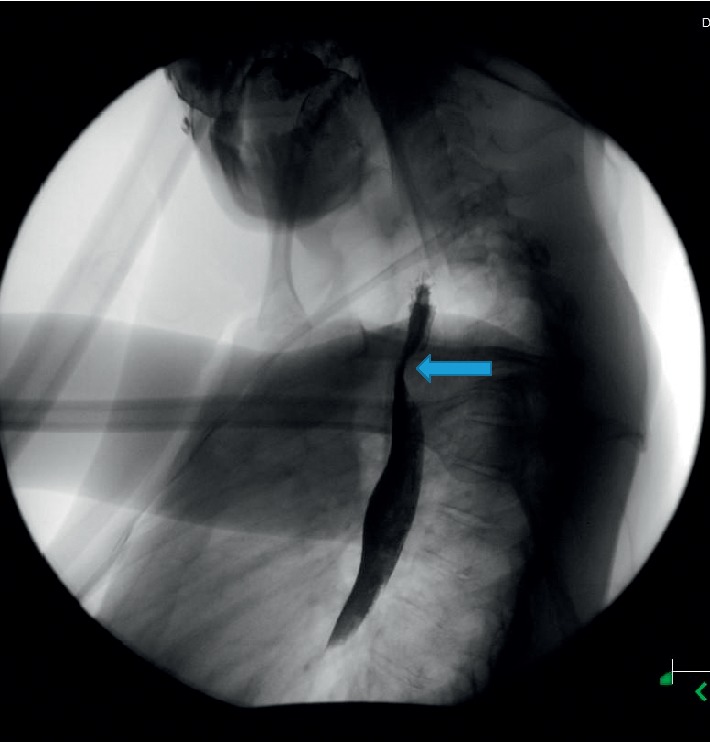
Esophagram with the upper esophageal compression.

**Figure 5:**
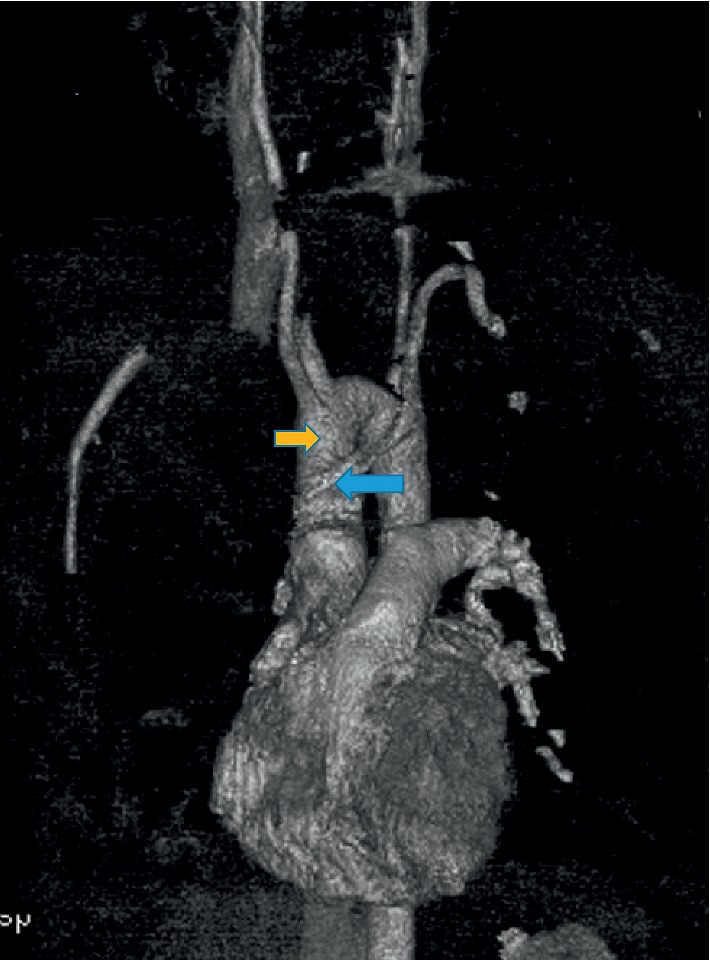
Type N1 morphology of the aberrant right subclavian artery (blue) arising from right side aortic arch (yellow) shown in 3D reconstruction of the computerized tomography-assisted angiography of the neck and chest.

A 31-year-old man with intellectual disability and cerebral palsy presented to the emergency department with recurrent esophageal food impaction. He had no medical history of asthma or food-related allergies. His family history and social history were otherwise unremarkable. He was allergic to phenobarbital medication with no clear details available about the allergic reaction. Physical examination including vital signs and abdominal and cardiorespiratory examination was within normal limits. His neurologic examination was notable for his inability to communicate, follow commands, or ambulate. The basic laboratory investigations including complete blood count (CBC), comprehensive metabolic panel (CMP), and coagulation profile were within normal limits except mild chronic microcytosis. There was no laboratory evidence of peripheral eosinophilia. The IgE-mediated allergic test was unremarkable. He underwent esophagogastroduodenoscopy (EGD) with upper and distal esophagus biopsy. He had an increased eosinophilic count of >15/hpf (Figure 1) in both biopsies and was diagnosed with eosinophilic esophagitis. He was initially managed with the proton pump inhibitor with persistent esophageal eosinophilia on repeat endoscopy. He was managed with oral 1 mg of budesonide (0.5 mg per ml repulse) two times a day for 6 weeks. The viscous solution was mixed with Splenda®. The patient's mother reported the compliance to the regimen, and he swallowed the viscous solution with no nausea or vomiting. He continued to have elevated eosinophils on repeat endoscopy despite steroid and elimination diet though the eosinophilic count was significantly decreased compared to previous esophageal biopsy (Figure 2). He was on oral budesonide during the index presentation to ER and had impaction of a respule. In recent hospital admission, he presented to the emergency department with esophageal foreign body impaction and underwent emergent endoscopy with retrieval of foreign bodies. During endoscopy, he was noted to have normal-appearing esophageal mucosa, proximal esophageal dilatation with extrinsic compression at the level of upper esophagus. To further delineate the cause of recurrent esophageal impaction, computed tomography (CT) scan of the neck and chest was performed which revealed an aberrant left subclavian artery arising from the right-sided aortic arch compressing the upper esophagus (Figures 3(a)–3(c)). Barium esophagogram reviewed compression in the upper esophagus (Figure 4). He was diagnosed with a rare type of dysphagia lusoria in setting of underlying eosinophilic esophagitis. His mother was instructed to continue steroid therapy and puree consistency diet. The patient was referred for surgical intervention. The CT angiogram of neck and chest was performed for the evaluation of vascular anatomy. The imaging studies were performed with the LightSpeed (GE®) 64 slice CT scanner. The three-dimensional reconstruction of the CT angiographic revealed type N-1 morphology of the aberrant right subclavian artery (Figure 5). However, the patient's mother refused surgical management of artery lusoria.

## 3. Discussion

Dysphagia lusoria is defined as difficulty in swallowing due to vascular compression of the esophagus by an aberrant right subclavian artery (artery lusoria). The prevalence of artery lusoria in the general population is 0.7% [5]. Compression of the esophagus is caused by course of the right subclavian artery from the left to the upper right-side posterior to the esophagus. In an endosonographic series of 1629 patients, the reported incidence of artery lusoria is 0.4% [6]. However, majority of them are asymptomatic [5]. Adachi–Williams' classification described four main morphologic types based on the anatomy for right subclavian, carotid truck, and the aortic arch (Table 1) [7]. The type N-1 morphology has a right aortic arch with left subclavian artery origin succeeding to both corotid arteries and the right subclavia. The type N-1 aberrant subclavian artery is rarest of all four morphology.

Cerebral palsy has been associated with the congenital heart disease [8]. The association of the aberrant origin of the right subclavian artery has been not been reported in patients with cerebral palsy. On the other hand, patients with the Edwards and Downs syndrome have high incidence of the aberrant right subclavian as compared to the general population [9]. The most common age of presentation for dysphagia lusoria is in the elderly age group. It presents as recurrent cough in the elder age group as compared to repeated chest infections in children [10]. In children, respiratory infections result due to compression of the soft and cartilage deprived trachea from the right aberrant subclavian artery. With age, the strengthening of the tracheal cartilage maintains the airway patency and the esophageal compression induced dysphagia remains the predominant presentation. The cough can be aspiration induced as well.

In a systematic study of 141 cases of dysphagia lusoria, with majority of them reported in the United States, coexistence of the right-sided aortic arch was present in 9.2% of patients [11]. It can be associated with the Kommerell diverticulum [12], which is a remnant of the left dorsal arch and can lead to dysphagia. In view of the unavailability of the contrast study, the evaluation remains limited in our scenario.

The initial evaluation includes the barium esophagogram to demonstrate the extrinsic compression by the aberrant right subclavian artery. The indication of the esophagogastroduodenoscopy is not clearly demonstrated [13], however frequently practiced. The esophageal motility study is not typical and has not shown to alter the management [10]. The vascular imaging studies are performed for the thorough evaluation and recognition of associated anomaly. Though there are no robust recommendations, the surgical intervention should be performed for the ones who are symptomatic and those with aneurysm [14]. All patients might not improve after the surgery [10]; hence, preoperative evaluation for the alternative etiology should be done as well.

There is an increase in incidence of the EoE over the last few decades [15, 16]. Certainly, this has increased the prevalence of EoE across the globe [17]. It is likely that eosinophilic esophagitis can present with other causes of dysphagia. There has been a recent update in the definition of EoE specifically focused on the better understanding of the proton pump inhibitor refractor esophageal eosinophilia (PPI-REE) [18]. It is suggested to evaluate for the secondary cause of the esophagus eosinophilia prior to concluding to diagnosis of the EoE [18, 19].

Increased number of eosinophils in the esophageal mucosa can be an isolated finding or in association with other secondary causes [4]. Isolated esophageal eosinophils occur mainly in three diseases, namely, eosinophilic esophagitis, gastroesophageal reflux disease, and proton pump inhibitors responsive esophageal eosinophilia. Several other causes, like achalasia, celiac disease, Crohn's disease, connective tissue disorders, and drug-induced hypersensitivity, may have increased esophageal eosinophils as a secondary finding, and diagnoses in such cases is based on other associated findings of the disease. It is noteworthy that increased esophageal eosinophils have also been reported with candida esophagitis resulting from prolonged stasis of food and secretions in the esophagus [20]. Our patient does meet histopathologic criteria for esophageal eosinophilia, that is, more than 15 eosinophils per high-power field. However, as per the current consensus, If extrinsic compression is considered to be a secondary cause of the esophageal eosinophilia, then our patient does not meet the criteria for the EoE [18, 19]. But the number of eosinophils in the esophageal biopsy may have also resulted from prolonged stasis of food and secretions, and it is not possible to assess how many eosinophils resulted due to this etiology.

The cerebral palsy posed a major challenge in the management of dysphagia in our case. The patient's inability to communicate and cognition deficit hamper the initial evaluation [21] and management. In a clinical scenario like ours, the clinical judgment guides the decision for the esophageal biopsy, and hence esophagogastroduodenoscopy was performed and esophageal biopsies of normal esophageal mucosa as per current standards were done.

In our case, it is difficult to assess which disease, eosinophilic esophagitis or dysphagia lusoria, is primarily responsible for the symptoms. It is likely that both etiologies are contributing towards long-term dysphagia; however, recent food impaction site being the upper esophagus suggests the most likely etiology to be dysphagia lusoria. Esophageal compression was in cervical esophagus, and esophageal eosinophilia was noted on the lower and middle esophagus. Complete normalization in esophageal eosinophils in response to topical steroids was seen in our case, but it remains a challenge for us to know if the patient still has symptoms due to unaddressed dysphagia lusoria.

## 4. Conclusion

Artery lusoria (aberrant right subclavian artery) is an unusual cause of dysphagia. The type N-1 morphology with the right-sided aortic arch is extremely rare. Etiology of the dysphagia is due to extrinsic compression. The food stasis due to compression can possibly lead to increased esophageal eosinophils. Association between eosinophilic esophagitis and dysphagia lusoria is rare and has not been reported before. However, in our case, it remains undetermined whether increased number of esophageal eosinophils resulted from primary eosinophilic esophagitis or due to prolonged stasis due to an aberrant subclavian artery.

## Figures and Tables

**Table 1:** The types of aberrant right subclavian artery.

Type G-1	The right subclavian artery arises as the last branch of the distal aorta. The right carotid, left carotid, and the left subclavian artery follow the normal trend

Type CG-1	The right subclavian artery is anomalous (as in type G) and the left vertebral artery originates from the aortic arch

Type H-1	The right subclavian artery is anomalous (as in type G), and both (right and left) carotid arteries arise from a common trunk named bicarotidic trunk

Type N-1 (as presented in our case)	This pattern is a mirror image of type G. There is a right aortic arch and the left subclavian artery origin succeeds both corotid arteries and the right subclavia. This is among the rarest of the aberrant left subclavian artery
